# A Comparison of the Efficacy of Surgical Renal Denervation and Pharmacologic Therapies in Post-Myocardial Infarction Heart Failure

**DOI:** 10.1371/journal.pone.0096996

**Published:** 2014-05-15

**Authors:** Jialu Hu, Yinliang Li, Wenbo Cheng, Zhen Yang, Fang Wang, Peng Lv, Conway Niu, Yuemei Hou, Yan Yan, Junbo Ge

**Affiliations:** 1 Department of Cardiology, Zhongshan Hospital, Fudan University, Shanghai, China; 2 Department of Cardiology, Shanghai Jiaotong University affiliated Sixth People's Hospital South Campus, Shanghai, China; 3 Department of Diagnostic Radiology, Zhongshan Hospital, Fudan University, Shanghai, China; 4 Shanghai Medical College, Fudan University, Shanghai, China; Tokai University, Japan

## Abstract

**Objective:**

Although renal denervation (RD) has been shown to be effective in treating post- myocardial Infarction (MI) heart failure (HF) in animal models and clinical trials, its utility as a standalone treatment without traditional drug treatment for post-MI HF still needs to be investigated.

**Methods:**

Rats were randomly assigned into seven experimental groups: N group (control group with no MI and no RD, n = 10), MI group (MI, n = 20), RD group (renal denervation, n = 10), RD-3d+MI group (RD performed three days before MI, n = 15), β-blocker-3d+MI group (Metoprolol treated three days before MI, n = 15), ACEI-3d+MI group (Perindopril treated three days before MI, n = 15), and ARB-3d+MI group (Losartan treated three days before MI, n = 15). Cardiac function, autonomic nervous system parameters, and neuroendocrine activities were evaluated 8 weeks post MI.

**Results:**

Compared to β-blockers, ACEIs, and ARBs, RD alone provided significantly better cardiac remodeling and function, enhanced water and sodium excretion, and improved autonomic modulation.

**Conclusions:**

In this post-MI HF animal model, surgical RD provides effective autonomic modulation, inhibition of the RAAS, improved cardiac remodeling, and preserved renal function, without affecting normal circulation and cardiopulmonary function in normal rats. Compared to β-blocker, ACEI, and ARB single-drug therapies, RD alone is more efficacious. These results suggest that RD may be an effective treatment option for HF, especially in patients who have contraindications to drug therapy.

## Introduction

Heart failure (HF) is a syndrome associated with high morbidity and mortality. Among all causes, myocardial infarction (MI) accounts for one of the most important. Although the advent of modern drug therapy and some non-drug treatments have provided symptomatic reliefs and improved survival rates in HF patients [1]–[3], many limitations remain. Alternative treatment modalities are needed in this patient population.

Radiofrequency renal denervation (RD) has been demonstrated in recent clinical trials to be effective in lowering blood pressure in patients with refractory hypertension [4], [5]. Possible mechanisms include altering both afferent and efferent renal sympathetic activity, thereby reducing excessive sympathetic activation and sodium and water retention [6], [7].

The effects of surgical RD intervention on post-MI HF were studied by Nozawa et al. and demonstrated that RD significantly attenuates LV remodeling, increases LV function, and increases excretion of sodium and water [8], With our previous study confirming these findings. Similar results were observed regardless of when RD was performed relative to MI: three days pre-MI or one day post-MI [9]. Davies et al. confirmed that RD improved both symptoms and exercise capacity in HF patients with no intra-procedural or post-procedural complications following RD [10].

Although RD has been shown to be effective in treating post-MI HF in animal models and clinical trials, its efficacy, as a stand-alone therapy, compared to the traditional pharmacotherapy for HF is unknown. In this study, we investigated the effects of surgical RD on cardiac function, autonomic nervous system parameters, and neuroendocrine activities as compared to β-blockers, ACEIs, and ARBs in a post-MI HF model.

## Methods

The experimental protocol and the use of animals were approved by the animal use and management ethics committee of the First Affiliated Hospital of Xinjiang Medical University. Experimental design and implementation were conducted in accordance with animal welfare guidelines, 3R principles, and AAALAC (Association for Assessment and Accreditation of Laboratory Animal Care) standards.

One hundred male Wistar rats (SPF level, 280–330 g) were housed in groups at a room temperature of 22–25°C with 50 to 70% humidity and a 12-hour light/dark cycle. A standard rat diet containing 0.3% NaCl and tap water were given ad libitum throughout the experimental period.

Rats were randomly assigned into seven experimental groups: N group (control group with no MI and no RD, n = 10), MI group (MI, n = 20), RD group (renal denervation, n = 10), RD-3d+MI group (RD performed three days before MI, n = 15), B-blocker-3d+MI group (Metoprolol treated three days before MI, n = 15), ACEI-3d+MI group (Perindopril treated three days before MI, n = 15), and ARB-3d+MI group (Losartan treated three days before MI, n = 15).

### Preparation for Post-MI HF Model

Wistar rats were injected intraperitoneally with ketamine (75 mg/kg), diazepam (5 mg/kg), and atropine (0.05 mg/kg). Tracheal intubation was connected to a ventilator. A thoracotomy was made at the anterolateral third intercostal space. Theleft anterior descending branch was then ligated. Rats that experienced ventricular fibrillation were injected intraperitoneally with 1% lidocaine (1 mg/kg). The thorax was then closed. Spontaneous respiration was restored and rats were placed in an environment at 28°C. Thirty minutes later, electrocardiography (ECG) was used to ensure that the model was successfully created with elevated ST segments. After the rats regained consciousness, they were injected intramuscularly with pethiadine once and penicillin twice daily for three days.

### Renal Denervation

After rats were anesthetized, a midline abdominal incision was made, and a cotton swab was used to open the renal adipose capsule. Saline-soaked sterile gauze was used to cover the intestines. The abdominal aorta and renal artery were separated, with the renal artery exposed at about 3 cm from the abdominal aorta. All visible renal nerve bundles were removed and adventitia of the renal artery and vein were stripped. Blood vessels were then gently painted with a cotton swab soaked with a solution of 95% ethanol and 10% phenol in ethanol. In selective rats, we evaluated the effects of RD by electrically stimulating the renal sympathetic nerves (Grass S48 nerve stimulator, 15 V, 0.2 ms, 10 Hz) at the proximal renal artery for 10–30 seconds before and after RD. In normal rats, electrical stimulation increased blood pressure by 5–10 mmHg, increased heart rate by 8–15 bpm, and caused the kidney to become paler in color. When electrically stimulated after RD, sympathetic effects were absent with no apparent changes in heart rate, blood pressure, or kidney color.

### Medication Treatment

Drugs were administered by oral gavage for eight weeks. Metoprolol (AstraZeneca pharmaceutical Co., Ltd, China) was administered twice daily (20 mg/kg/day) to rats in the β-blocker-3d+MI group. Perindopril (Shi Wei Ya Pharmaceutical Co., Ltd, China) was administered once daily (3 mg/kg/day) to rats in the ACEI-3d+MI group. Losartan (MSD pharmaceutical Co. Ltd, China) was administered once daily (20 mg/kg/day) to rats in the ARB-3d+MI group.

### Measurement of Urine Volume and Sodium Excretion

The rats were moved to individual metabolic cages with free access to tap water eight weeks post-MI. The acclimatization period was designed to be two days, with urine collected beginning in the third 24-hour period. After the measurement of urinary volume, the rats were returned to their original cages. Urine sodium concentration was measured by electrolyte analyzer IMS-972Plus (Xi Cai Heng Medical Electronics, China), using the ion-selective electrode method.

### Measurement of Left Ventricular Function

Left ventricular (LV) function was determined with echocardiography eight weeks post-MI. Echocardiography was performed under ether anesthesia with a high-resolution small animal echocardiographic system equipped with a Heart RMV710 probe (Visual sonic, Toronto, Canada). A two-dimensional short-axis view of the LV was obtained at the level of the papillary muscle along with the M-mode recordings. End-diastolic and end-systolic LV endocardial dimensions (LVESV, LVEDV), fractional shortening (FS) and ejection fraction (EF) were determined from at least three consecutive cardiac cycles.

### Determination of Heart Rate and Heart Rate Variability

At eight week post-MI, continuous 6-lead ECG was recorded for 5 minutes. Using Lab-Chart version 6.1.3 for Windows (AD Instruments, Sydney, Australia), heart rate (HR) and heart rate variability (HRV) were calculated. Time-domain analysis included measurement of the mean R-R interval during sinus rhythm and its standard deviation (SDNN).

### Measurement of Plasma Renin, Angiotensin II, Aldosterone, BNP, Endothelin and Norepinephrine Levels

After the hemodynamic study was completed, rats were weighed, and blood was collected directly from the heart via the thoracotomy incision. Plasma renin, angiotensin II, aldosterone, and BNP levels were measured by radioimmunoassay (test kit manufactured by Phoenix Pharmaceuticals, USA). Plasma norepinephrine (NE) was determined by the HPLC-electrochemical method. Plasma endothelin-1 (ET-1) was determined by ELISA (test kit manufactured by Immuno-Biological Laboratories Co., Ltd, Germany).

### LV infarct size determination

After blood sampling, the heart was removed and washed with saline, filter dried, and then weighed with an electronic scale. The LV, including the ventricular septum, was dissected, separated, and weighed. Incisions were made in the LV so that its tissue could be flattened. The LV circumference and the area of infarction were outlined on a clear plastic sheet for both endocardial and epicardial surfaces [11]. Rats with an LV infarct size of 30% or greater were used for data analysis in this study.

### Statistical Methods

Each datum conformed to normal distribution. Group comparisons were made with analysis of variance (ANOVA), followed by the Bonferoni test (least significant difference, LSD) to identify differences among groups. Results were expressed as mean ± SD. A value of P<0.05 was considered statistically significant.

## Results

Ten of 20 animals in the MI group, 5 of 15 in the RD-3d+MI group, 5 of 15 in the β-blocker-3d+MI group, 7 of 15 in the ACEI-3d+MI group and 8 of 15 in the ARB-3d+MI group died within the eight week period after coronary artery ligation. The death rates of the RD-3d+MI group and the β-blocker group were the same and much less than the MI, ACEI, or ARB groups (P<0.05). The death rate did not differ between the latter three groups. None in the control and RD group died during the experiment. There were no significant differences in body weight or the infarct size among all experimental groups eight weeks post-MI (Table 1).

**Table 1:** Rat weight, infarct ratio, BNP, urinary volume, and urinary sodium eight weeks post-MI.

Group (n = Samples/Total)	Weight (g)	LV infarct ratio (%)	Urine volume (ml/day)	Urinary Na^+^ (mmol/day)	BNP (pg/ml)
N (n = 10/10)	410±42	/	22±4.9	0.25±0.04	82±12
MI (n = 10/20)	420±36	35±5.6	11±3.8*	0.11±0.03*	490±40*
RD(n = 10/10)	415±38	/	23±5.9#	0.26±0.06#	86±10#
RD-3d-pre-MI (n = 10/15)	402±55	32±4.4	20±3.3#	0.23±0.05#	130±15* #
β-blocker-3d-pre-MI (n = 10/15)	422±47	34±3.4	13±3.6* †	0.14±0.04* †	263±22* ^#^ †
ACEI-3d-pre-MI (n = 8/15)	419±33	36±6.5	13±4.7* †	0.13±0.03* †	241±26* ^#^ †
ARB-3d-pre-MI (n = 7/15)	417±59	33±6.4	10±5.1* †	0.14±0.03* †	248±28* ^#^ †

N: Normal group; RD: renal denervation; MI: myocardial infarction. Data are means ± SD obtained four weeks post-MI.

*P<0.05 vs. N;

# P<0.05 vs. MI;

† P<0.05 vs. RD-3d-pre-MI;

β-blocker: metoprolol

ACEI: perindopril

ARB: irbesartan

### Changes in Urine Volume, Urinary Sodium and BNP

Compared with the normal (N) group, urine volumes of the MI, β-blocker, ACEI and ARB groups were all significantly lower (P<0.05), while there was no significant reduction in the RD and RD-3d+MI group compared to the normal group. Urinary sodium levels also followed a similar trend (Table 1). The serum BNP level was significantly increased in the MI group with no change in the RD group. While serum BNP was also increased in the RD-3d+MI group, it was significantly less elevated compared to the β-blocker group, ACEI group and ARB group (Table 1).

### Changes in LV Function

There was significant LV remodeling in the MI group with marked decreases in EF and FS indicating a reduction in LV systolic function eight weeks post MI. Although EF and FS were both lower and the LVEDV and LVESV were both higher in the RD-3d-pre-MI group, compared to the normal group (P<0.05), they were all significantly improved compared to the MI group (P<0.05). The EF, FS, LVEDV and LVESV in the other three drug-only groups also showed improvement over the MI group (P<0.05), but results were not as profound as the RD-3d+MI group and with no marked difference between them. There was no difference between the RD and normal groups (P>0.05) (Table 2).

**Table 2:** LV function in rats eight weeks post-MI.

Group (n = Samples/Total)	LVEF (%)	LVFS (%)	LVEDV (ml)	LVESV (ml)
N (n = 10/10)	87±4.2	52±5.6	0.72±0.19	0.1±0.04
MI (n = 10/20)	37±6.7*	16±3.6*	2.16±0.68*	1.37±0.04*
RD(n = 10/10)	83±6.7#	49±4.3#	0.71±1.09#	0.15±0.09#
RD-3d-pre-MI (n = 10/15)	73±5.5* #	40±4.4* #	0.75±0.19#	0.2±0.08* #
β-blocker-3d-pre-MI (n = 10/15)	52±4.7* ^#^ †	34±3.4* ^#^ †	0.83±0.16#	0.40±0.14* ^#^ †
ACEI-3d-pre-MI (n = 8/15)	49±7.3* ^#^ †	31±6.5* ^#^ †	0.85±0.16#	0.43±0.07* ^#^ †
ARB-3d-pre-MI (n = 7/15)	47±5.9* †	30±6.4* ^#^ †	0.89±0.11#	0.46±0.08* ^#^ †

N: Normal group; RD: renal denervation; MI: myocardial infarction. Data are means ± SD obtained four weeks post-MI.

*P<0.05 vs. N;

# P<0.05 vs. MI;

† P<0.05 vs. RD-3d-pre-MI;

β-blocker: metoprolol

ACEI: perindopril

ARB: irbesartan.

### Changes in Plasma Renin, Angiotensin II, Aldosterone and NE Levels

**Figure 1:**
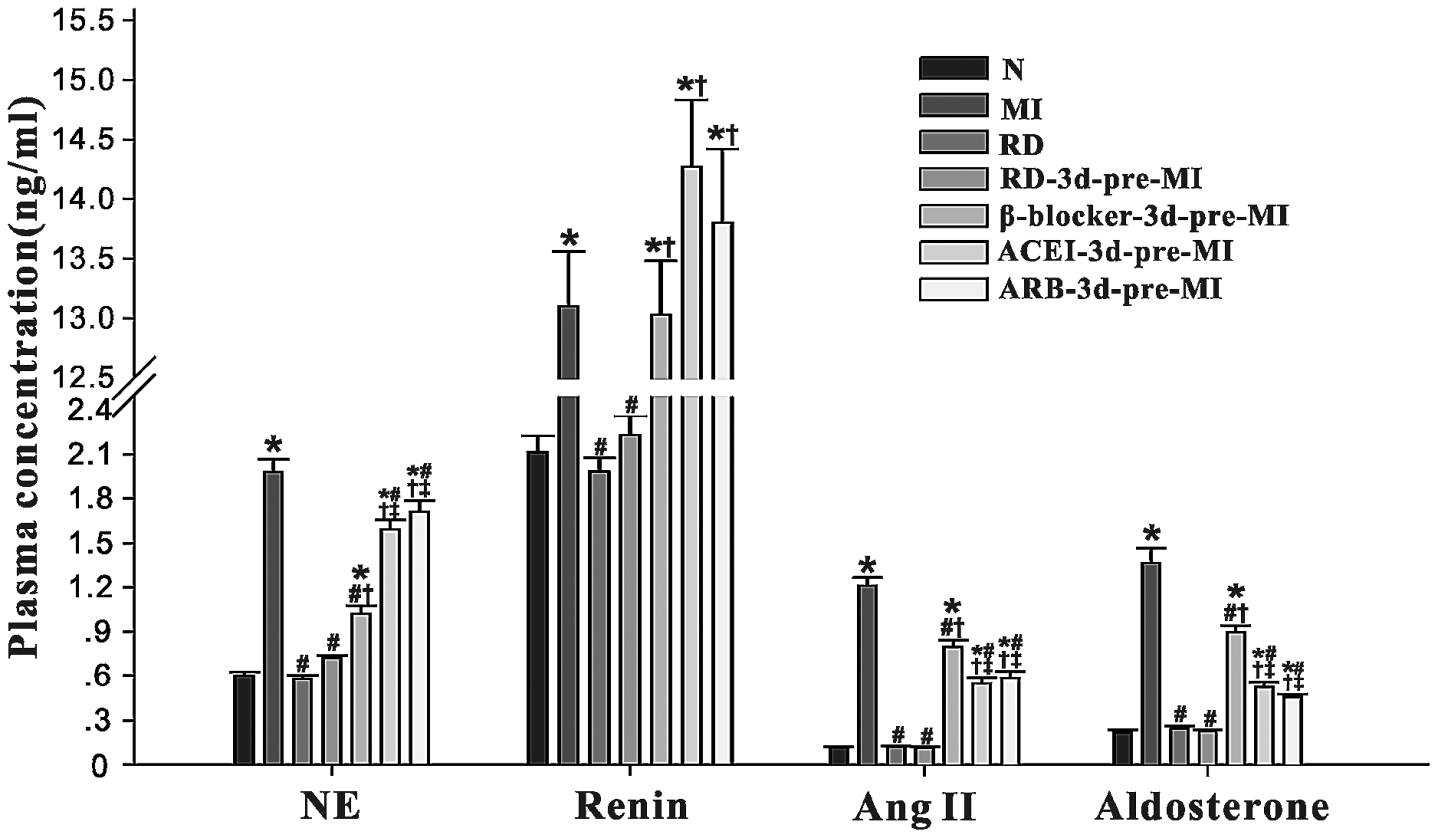
Changes in plasma norepinephrine (NE), renin, angiotensin II, and aldosterone levels in rats eight weeks post-MI. N: Normal group; RD: renal denervation; MI: myocardial infarction. Data are means ± SD obtained four weeks post-MI. * P<0.05 vs. N; # P<0.05 vs. MI; † P<0.05 vs. RD-3d-pre-MI; ‡ P<0.05 vs. β-blocker-3d-pre-MI. β-blocker: metoprolol; ACEI: perindopril; ARB: irbesartan.

Compared with the normal group, plasma renin, angiotensin II, aldosterone and NE levels were all significantly increased in the MI group (P<0.05) eight weeks post MI, with no significant change in the RD and RD-3d+MI groups (P>0.05). The drug only groups (β-blocker, ACEI and ARB groups) showed modest and intermediate changes in these autonomic and neuroendocrine parameters. Among the three drugs, β-blockers showed a better effect in lowing NE levels while ACEIs and ARBs were better in suppressing the RAAS. RD seemed to have had no effect on normal rats (Figure 1).

### Changes in HRV

**Figure 2:**
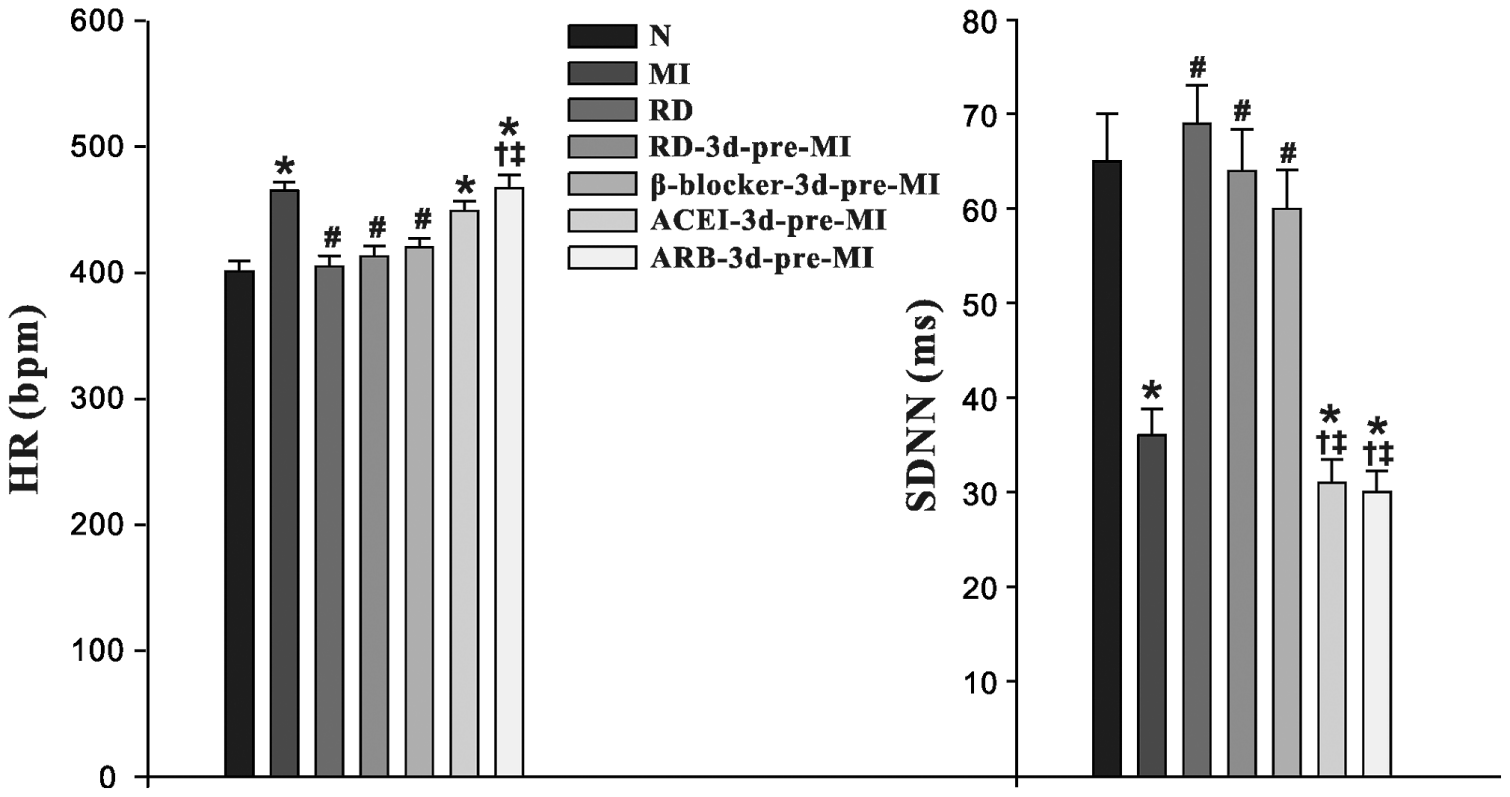
Effects of RD on HR and SDNN in rats eight weeks post-MI. N: Normal group; RD: renal denervation; MI: myocardial infarction. Data are means ± SD obtained four weeks post-MI. * P<0.05 vs. N; # P<0.05 vs. MI; † P<0.05 vs. RD-3d-pre-MI; ‡ P<0.05 vs. β-blocker-3d-pre-MI. β-blocker: metoprolol; ACEI: perindopril; ARB: irbesartan.

Compared with the normal group at eight weeks, the MI, ACEI and ARB groups all had significantly increased HR (P<0.05), decreased SDNN (P<0.05), indicating dominant cardiac sympathetic activation. There was no significant change in the RD-3d+MI, β-blocker and RD groups compared with the normal group (P>0.05), suggesting that over-activation of the cardiac sympathetic nervous system was suppressed by RD and β-blockers in post-MI HF rats, and that RD had no effect on normal rats (Figure 2).

## Discussion

This parallel controlled study compared the effects of conventional drug treatment for HF (β-blocker, ACEI and ARB) with stand-alone RD in a post-MI HF animal model. The results showed that the physiologic benefits of RD on improving cardiac remodeling and function, water and sodium excretion, autonomic modulation and suppression of RAAS activation were significantly better than any of the three drugs alone and had no effect on normal controls.

### Comparison of efficacy of RD vs. β-blocker

β-blockers are the cornerstone in HF therapy, and are among the few treatment options associated with long-term improvements in outcome. However, its use entails cardio-selectivity, hemodynamic tolerance and safety issues. In contrast to β-blockers, RD does not affect the cardiac autonomic nervous system (CANS) directly. However, RD results in a similar effect on HRV as β-blockers with decreased HR and increased SDNN, indicating suppression of cardiac sympathetic activation which, theoretically, helps in the occurrence of malignant arrhythmias in post-MI HF [12]. The effect of RD on the CANS may be due to the blockade of the renal sympathetic afferent nerve, which inhibits the central nervous system, thereby indirectly affecting the heart.

In addition, RD results in a substantially better restoration of plasma NE levels in HF compared to β-blockers alone. This reflects a significant reduction in the central sympathetic tone, likely attributed to the simultaneous inhibition and suppression of the RAAS. RD also produces marked improvement in water and sodium excretion, along with reduction of BNP level, resulting in greater physiologic benefit than β-blockers.

In this study, RD-3d+MI and the β-blocker groups had similar survival rates eight weeks post MI and were significantly better than the other groups. In patients with refractory hypertension, renal sympathetic denervation did not result in acute cardiac decompensation [13]. All these results suggested that RD only alleviates renal nerves' input to the abnormal cardiovascular responses in the setting of HF, while not interfering with normal cardiovascular physiology. RD may provide a more efficacious, safer treatment option than β-blockers in patients with HF, with fewer contraindications.

### Comparison of RD and RAAS-inhibiting drugs

In chronic HF, renal sympathetic activity is increased, activating β1 receptors and increasing renin release. This then stimulates the RAAS and the α1 receptors, causing renal artery contraction, and reducing renal blood flow [1]-[2]. RD inhibits efferent renal sympathetic activities, suppresses the RAAS and improves renal perfusion. Our findings showed that RD indeed caused a decrease in plasma renin, angiotensin II, and aldosterone levels. When comparing RD to ACEI or ARB treated groups, death rates in HF rats were significantly lower (P<0.05), with greater inhibition of the sympathetic nervous system (SNS) and RAAS activation, resulting in improved cardiac remodeling. The superior physiologic benefits of RD can be attributed to concurrent inhibition of the SNS, improved renal blood supply, and increased water and sodium excretion. Clinically, the use of ACEIs and ARBs may be contraindicated in HF patients with severe renal insufficiency. RD provides a feasible alternative in the treatment of HF, with renal protection and possibly avoiding side effects from bradykinin inhibition, such as coughing.

### Limitations

Limitations of the study include a relatively short follow up observation period, along with the possibility of renal nerve regeneration. In our previous study, we observed MI rats one month post-RD. Considering renal nerve regeneration and other potential variables, we extended our observation to two months post-RD in this study and demonstrated persistent efficacy of RD. Future studies are required to determine the long term effects of autonomic modulation and clinical applications of RD in patients with HF.

Another study limitation is the single drug treatment model. Clinical practice protocols in HF management often entail a multi-drug regimen. Further studies comparing RD to multi-drug pharmacotherapy are needed.

## Conclusions

In this post-MI HF animal model, surgical RD provides effective autonomic modulation, inhibition of the RAAS, improved cardiac remodeling, and preserved renal function, without affecting normal circulation and cardiopulmonary function in normal rats. Compared to β-blocker, ACEI, and ARB single-drug therapies, RD alone is more efficacious. These results suggest that RD may be an effective treatment option for HF, especially in patients who have contraindications to drug therapy.
